# Numerical Investigation of a Novel Wiring Scheme Enabling Simple and Accurate Impedance Cytometry

**DOI:** 10.3390/mi8090283

**Published:** 2017-09-18

**Authors:** Federica Caselli, Riccardo Reale, Nicola Antonio Nodargi, Paolo Bisegna

**Affiliations:** Department of Civil Engineering and Computer Science, University of Rome Tor Vergata, Via del Politecnico 1, 00133 Rome, Italy; Real88@hotmail.it (R.R.); nodargi@ing.uniroma2.it (N.A.N.)

**Keywords:** microfluidics, electrical impedance, single-cell analysis, lab-on-a-chip, finite element method, virtual laboratory

## Abstract

Microfluidic impedance cytometry is a label-free approach for high-throughput analysis of particles and cells. It is based on the characterization of the dielectric properties of single particles as they flow through a microchannel with integrated electrodes. However, the measured signal depends not only on the intrinsic particle properties, but also on the particle trajectory through the measuring region, thus challenging the resolution and accuracy of the technique. In this work we show via simulation that this issue can be overcome without resorting to particle focusing, by means of a straightforward modification of the wiring scheme for the most typical and widely used microfluidic impedance chip.

## 1. Introduction

Microfluidic impedance cytometry is a label-free technique for analysing single particles and cells at high throughput [1,2]. It is used in different biological assays, including particle sizing and counting, cell phenotyping, and disease diagnostics (e.g., [3,4,5,6]). In a typical impedance chip, suspended particles flow through a microchannel and two electrode pairs are used to measure the variation in channel impedance induced by the passage of a particle. The impedance change is exploited to characterize particle properties, in addition to particle counting. As an example, information on the size, cell membrane, and intracellular conductivity of biological cells are obtained, depending on the frequency of the stimulating AC voltage [7]. However, like in a Coulter volume measurement, the recorded signal depends not only on the intrinsic properties of the particle, but also on its trajectory through the channel [8]. This is due to the non-uniformity of the electric field in the sensing region, and produces blurring of measured particle properties [9,10,11], thus challenging the accuracy and the resolution of the technique.

Great effort has been devoted to overcoming or mitigating this issue. In particular, electrode designs that reduce the non-uniformity of the electric field (e.g., [12,13]), as well as particle focusing mechanisms (e.g., [14,15]) have been proposed. However, the former cannot completely remove the positional dependence, whereas the latter increase the complexity of the system. Recently, we proposed a method to correct the measured particle properties via a simple compensation procedure [16,17]. That method hinges on the use of a metric encoding particle trajectory, obtained by exploiting either five pairs of facing electrodes [16] or five coplanar electrodes [17]. However, multi-electrode approaches require a larger sensing region, thus increasing the number of particle coincidences for a given sample concentration [18].

The aim of this work is to demonstrate in silico that the standard and simple impedance chip comprising two electrode pairs can also provide a metric encoding particle trajectory which is effective in solving the positional dependence issue. This is achieved by means of a straightforward change in the wiring scheme proposed in Reference [19] with respect to the usual and widely used operation mode. No particle focusing systems are required, and the idea applies to both the facing and coplanar electrode configurations.

The method is presented and validated by exploiting a virtual laboratory based on the finite element method. In particular, an in silico particle sizing experiment is performed, showing excellent discrimination performance of both designs. In addition, it is shown that, contrary to the conventional one, the novel wiring scheme yields an accurate estimate of particle velocity. The relationship among estimated particle size, velocity, and cross-sectional position is also studied. The numerical investigation performed in this paper is instrumental to have a clear and deep understanding of the related experimental activity. The latter is reported in the companion paper [19], and closely agrees with the present simulation study.

## 2. Operating Principle

**Figure 1:**
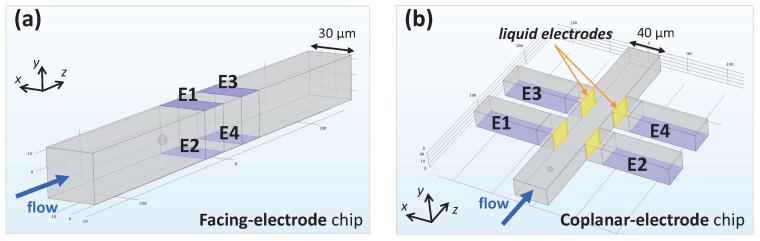
(**a**) Geometric model of a typical microfluidic impedance chip comprising two pairs of facing electrodes (E1–E2 and E3–E4 pairs). Dimensions are those of a commercial impedance chip by Micronit: 30 μ m × 28 μ m cross-section ( W×H in xy -plane), 20 μ m electrode width and spacing ( ||z ); (**b**) Geometric model of an impedance chip with coplanar electrode configuration: two pairs of *liquid* electrodes [22,23] are generated by two pairs of metal electrodes (E1–E2 and E3–E4 pairs). Dimensions are: 40 μ m × 21.5 μ m cross-section ( W×H in xy -plane), 30 μ m lateral channels width and spacing ( ||z ), 20 μ m electrode recess with respect to the main channel ( ||x ).

**Figure 2:**
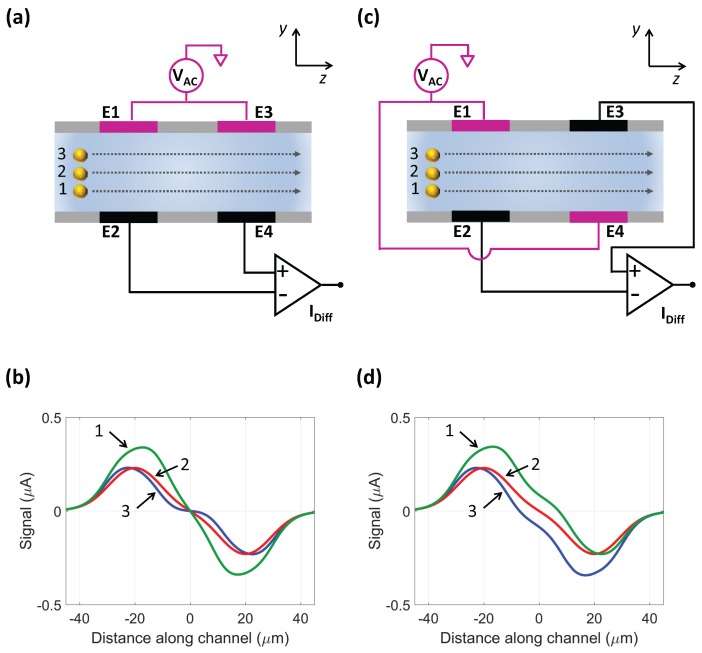
Facing electrode chip. (**a**,**c**) Side schematic view. Three particle trajectories are considered: in the lower half of the channel (trajectory 1), along the channel axis (trajectory 2), and in the upper half of the channel (trajectory 3). In (**a**) the conventional measuring scheme is shown: an AC voltage is applied to the top electrodes (E1 and E3) and the differential current flowing through the bottom (virtual ground) electrodes (E2 and E4) is measured. In (**c**) the novel measuring scheme is presented: an AC voltage is applied to diagonally opposite electrodes (E1 and E4), and the differential current flowing through the remaining (virtual ground) electrodes (E2 and E3) is measured; (**b**,**d**) Signal traces (finite element simulations) respectively recorded using the wiring schemes in (**a**,**c**). Insulating beads (6 μ m diameter) traveling along trajectories 1, 2, or 3 shown in panels (**a**,**c**) are considered (5 μ m gap from channel floor (respectively, ceiling) in trajectory 1 (respectively, 3)).

The geometric model of a standard microfluidic impedance chip with two pairs of facing electrodes is shown in Figure 1a, and its conventional wiring scheme is shown in Figure 2a. An AC voltage is applied to the top stimulating electrodes (E1 and E3), and the differential current flowing through the bottom measuring electrodes (E2 and E4) is collected. The use of a differential measurement scheme instead of an absolute one increases the signal-to-noise ratio and reduces the effect of electrode polarization [7]. The passage of a flowing particle is recorded as a pair of opposite pulses with the same amplitude. In fact, the recorded trace exhibits mirror symmetry with respect to the center of the sensing region [20,21], and the differential current is maximal (respectively, minimal) when the particle is approximately at the center of the E1–E2 (respectively, E3–E4) electrode pair (Figure 2b, curve 2). Pulse amplitude measured at low-frequency stimulating AC voltage is a measure of particle volume [1]. However, the electric field within the channel is non-uniform, and therefore the magnitude of the measured electrical current depends on particle trajectory height (i.e., *y*-coordinate of the center of the flowing particle). A further contribution to pulse amplitude comes from the cross current between the left and right pairs of electrodes (i.e., current flowing through a measuring electrode, E2 or E4, originating from the diagonally opposite stimulating one, respectively E3 or E1). As a consequence of the cross current, pulse amplitude is higher when the particle flows closer to the measuring electrodes than to the stimulating ones (Figure 2b, curves 1 and 3) . Therefore, the system is asymmetric top to bottom [10].

The novel wiring scheme investigated in this work is shown in Figure 2c: the AC voltage is applied to diagonally opposite stimulating electrodes (E1 and E4), and the differential current flowing through the remaining measuring electrodes (E2 and E3) is measured. In this way, a particle flowing in the lower (respectively, upper) half of the channel (Figure 2c, trajectory 1 (respectively, 3)) is located closer (respectively, farther) to the bottom measuring electrode E2 than to the top stimulating one E1 when detected by the left pair of electrodes. Then, it is located farther (respectively, closer) to the top measuring electrode E3 than to the bottom stimulating one E4 when detected by the right pair of electrodes. Accordingly, the left (respectively, right) pulse of the recorded signal is expected to have higher amplitude than the right (respectively, left) pulse (Figure 2d, curve 1 (respectively, curve 3)). Particles traveling through the middle of the channel produce pulses with equal amplitude (Figure 2d, curve 2). This suggests that (i) the average of pulse amplitudes is not affected by top–bottom asymmetry (i.e., it is the same for trajectories 1 and 3 in Figure 2c), thus yielding a better estimate of particle volume than either single pulse amplitude; and (ii) the difference of pulse amplitudes encodes particle trajectory height. In fact, the relative difference of pulse amplitudes (i.e., pulse amplitude difference divided by pulse amplitude average) is here chosen in order to obtain a metric independent of particle size.

**Figure 3:**
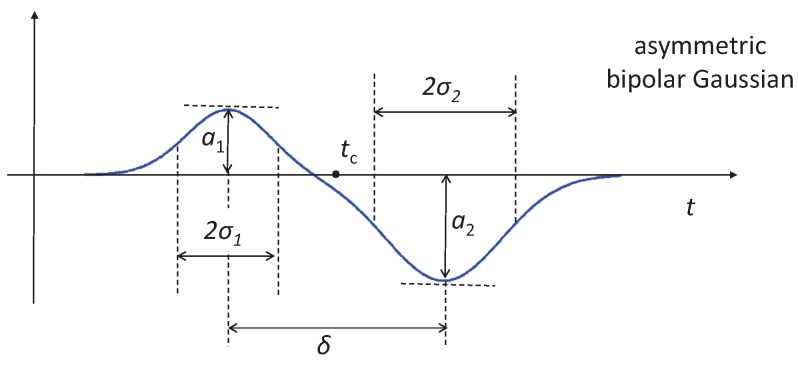
Asymmetric bipolar Gaussian template fitted to the differential current traces recorded when using the proposed wiring scheme.

An asymmetric bipolar Gaussian template (Figure 3) can be conveniently fitted to signal traces (Figure 2d) in order to extract the left- and right-pulse amplitudes. The template is obtained as the difference of two shifted Gaussian pulses with different amplitude and width, as follows:

(1) $$s\left(t\right)=g_{1}\left(t-t_{c}+\delta /2\right)-g_{2}\left(t-t_{c}-\delta /2\right),$$

with:

(2) $$g_{1}\left(t\right)=a_{1}e^{-t^{2}/\left(2\sigma_{1}^{2}\right)},\;g_{2}\left(t\right)=a_{2}e^{-t^{2}/\left(2\sigma_{2}^{2}\right)}.$$

This template depends on the following parameters: central time moment, $t_{c}$ ; transit time, δ ; pulse width controls, $\sigma_{1}$ and $\sigma_{2}$ ; pulse amplitude controls, $a_{1}$ and $a_{2}$ .

The cube root of the mean value of the pulse amplitude controls, $(a_{1}+a_{2})/2$ , yields an estimate *D* of the particle diameter *d*:

(3) $$D=G{\left[\left(a_{1}+a_{2}\right)/2\right]}^{1/3},$$

where *G* is a gain factor depending on chip geometry, buffer conductivity, and electrode double-layer impedance. However, the estimate *D*—referred to as “electrical diameter” in the following—suffers from a mild positional dependence issue, turning out to be greater for trajectories 1 and 3 than for trajectory 2 (Figure 2d).

The pulse amplitude relative difference Δ is introduced:

(4) $$\Delta =\frac{a_{2}-a_{1}}{(a_{1}+a_{2})/2}.$$

It is shown in Section 4 that Δ provides an electrical estimate *Y* of actual particle trajectory height *y*:

(5) Y=αΔH,

where *H* is channel height, α is a calibration coefficient depending on chip geometry, and the origin of the *y*-axis is chosen on the channel axis. The estimate *Y* is referred to as “electrical height” in the following. Moreover, the metric Δ is exploited to compensate for the spread in *D* induced by the positional dependence issue, thus obtaining a “corrected electrical diameter” D−corr (Equation (9)).

By comparison, in the conventional operation mode, a symmetric bipolar Gaussian template is used [20] (i.e., $a_{1}=a_{2}=a$ and $\sigma_{1}=\sigma_{2}=\sigma$ are enforced in Equation (1)). The electrical diameter *D* is computed by means of Equation (3), but the pulse amplitude relative difference Δ vanishes, not conveying additional information.

Using either wiring scheme, the transit time δ yields an estimate of the particle velocity *v* [16,24], namely the “electrical velocity”:

(6) V=L/δ,

where *L* is the peak-to-peak distance of signal traces (Figure 2b,d), approximated by the centre-to-centre spacing of the electrodes. Comparing the simulation traces in Figure 2d with those in Figure 2b, it appears that the peak-to-peak distance of traces provided by the new wiring scheme is less sensitive to particle trajectory than the one provided by the conventional wiring scheme. In fact, as shown in Section 4, the novel wiring scheme yields a more accurate estimate of particle velocity than the conventional wiring scheme.

The conventional and new wiring schemes discussed above can also be implemented in the microfluidic impedance chip with coplanar electrode configuration shown in Figure 1b. The field is generated by two pairs of lateral metal electrodes (E1–E2 and E3–E4), and is guided along access channels to the active area in the main channel. The equipotential surfaces at the apertures of the access channels behave as vertical *liquid* electrodes injecting the current into the main channel [22,23].

Using the conventional measuring scheme, the AC voltage is applied to stimulating electrodes E1 and E3, whereas using the new wiring scheme the AC voltage is applied to stimulating electrodes E1 and E4. In both cases, the differential current flowing through the remaining electrodes is measured. The electric field in the sensing region is nearly uniform along the channel height *y*. However, fringing field effects occur in the xz -plane and therefore the measured traces depend on particle lateral position *x* [23]. Using the new wiring scheme, the relative difference Δ between the amplitude of the opposite pulses of the signal trace is defined according to Equation (4), and turns out to encode the particle lateral position *x*. Accordingly, the “electrical position” *X* is given by:

(7) X=βΔW,

where *W* is channel width, β is a calibration coefficient depending on chip geometry, and the origin of the *x*-axis is chosen on the channel axis. The metric Δ can therefore be used to compensate for the spread in signal amplitude induced by the particle lateral position *x*.

## 3. Materials and Methods

The effectiveness of the proposed wiring scheme is shown by a numerical campaign, using a virtual laboratory that provides close-to-experimental synthetic data streams [25]. The following particle-sizing experiment is simulated: a mixture of insulating beads of 5, 6, and 7 μ m diameter (with size coefficients of variation CV = 2.5%, 1%, and 1%, respectively) is pumped through the virtual microfluidic chip assuming uniform distribution of particle centres across the channel cross-section. Both the facing electrode ( F ) and coplanar electrode ( C ) chips are considered, using both the conventional ( $\cdot^{\mathrm{conv}}$ ) and the new ( $\cdot^{\mathrm{new}}$ ) wiring scheme. The relevant synthetic data streams are denoted as follows: $F_{\mathrm{mix}}^{\mathrm{conv}}$ , $F_{\mathrm{mix}}^{\mathrm{new}}$ , $C_{\mathrm{mix}}^{\mathrm{conv}}$ , $C_{\mathrm{mix}}^{\mathrm{new}}$ .

**Figure 4:**
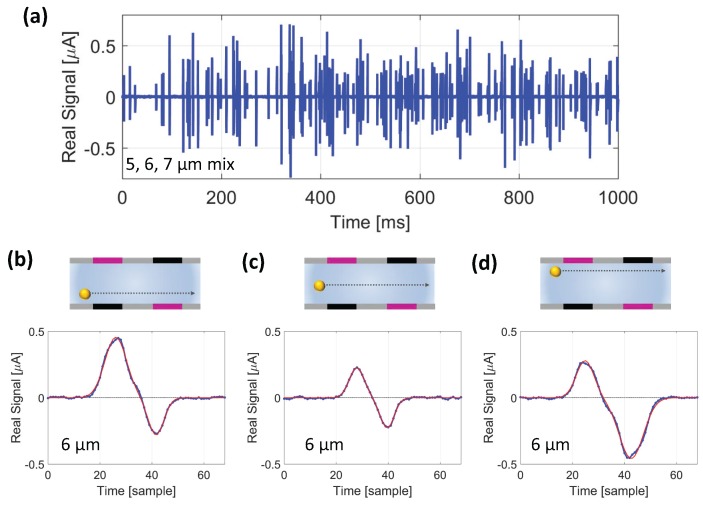
Facing electrode chip operated using the novel wiring scheme. (**a**) Portion of the synthetic data stream $F_{\mathrm{mix}}^{\mathrm{new}}$ , relevant to a mixture of 5, 6, and 7 μ m beads. (**b**–**d**) Exemplary events (blue curves) taken from the data stream in (**a**) and relevant to 6 μ m diameter beads traveling (**b**) close to the bottom of the channel, (**c**) through the middle of the channel, and (**d**) close to the top of the channel. Fitting templates are also shown (red curves).

Full details of the data streams’ generation are provided in Appendix A. Figure 4 shows one second of the syntectic data stream $F_{\mathrm{mix}}^{\mathrm{new}}$ , along with the zoom of three exemplary events.

The synthetic data streams were processed using an in-house software toolbox. First, event detection in the data stream was performed. Then, for each detected event, template fitting and feature extraction were carried out as follows. The asymmetric bipolar Gaussian template in Equation (1) was fitted to the differential signal traces recorded using the new wiring scheme. The electrical diameter *D*, electrical velocity *V*, pulse amplitude relative difference Δ , electrical height *Y* (facing electrode chip) or electrical position *X* (coplanar electrode chip) were computed and compared with the actual particle properties (diameter *d*, velocity *v*, cross-section coordinate *y* or *x*). The histogram of the root mean squared error of the fit, normalized by the mean value of the pulse amplitude controls, $(a_{1}+a_{2})/2$ , is reported in Figure S1 of Supplementary Materials. On the other hand, the symmetric bipolar Gaussian template—obtained as a particular case of the asymmetric one—was fitted to the data streams recorded using the conventional wiring scheme. In that case, only the electrical quantities *D* and *V* are defined.

## 4. Results and Discussion

**Figure 5:**
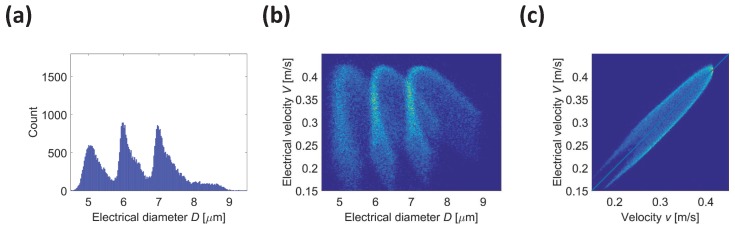
Facing electrode chip operated using the conventional wiring scheme. Virtual experiment relevant to a mixture of insulating beads with 5, 6, and 7 μ m diameter (data stream $F_{\mathrm{mix}}^{\mathrm{conv}}$ ). (**a**) Histogram of the electrical diameter *D*; (**b**) Density plot of electrical velocity *V* vs. electrical diameter *D*; (**c**) Density plot of electrical velocity *V* vs. velocity *v*.

Figure 5a shows the histogram of the electrical diameter *D* obtained using the conventional wiring scheme. The three bead populations (5, 6, and 7 μ m diameter) partially overlap and are not clearly distinguishable. Three main peaks are present, but there is a significant spread and skewness. The three populations cannot be separated even by jointly using the electrical diameter *D* and the electrical velocity *V* (Figure 5b). Moreover, Figure 5c shows that the electrical velocity *V* is not an accurate estimate of the actual particle velocity *v* (correlation coefficient 0.90). In fact, the peak-to-peak distance *L* of signal traces used in Equation (6) depends on particle trajectory height (cf. curve 1 and 3 in Figure 2b), and thus the electrode pitch may be a poor approximation of *L* for off-centre particles. Therefore, a positional dependence of the electrical velocity *V* appears.

**Figure 6:**
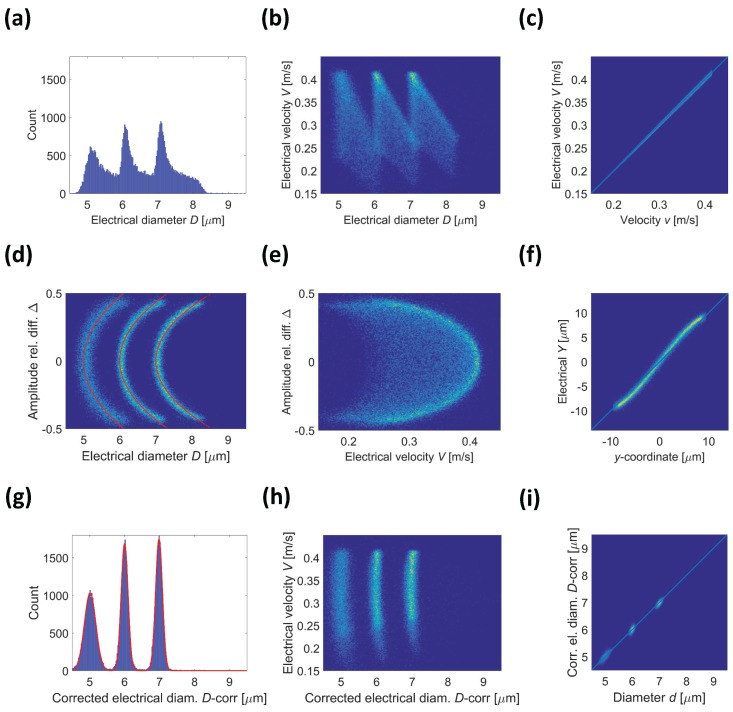
Facing electrode chip operated using the new wiring scheme. Virtual experiment relevant to a mixture of insulating beads with 5, 6, and 7 μ m diameter (data stream $F_{\mathrm{mix}}^{\mathrm{new}}$ ). (**a**) Histogram of the electrical diameter *D*; (**b**) Density plot of electrical velocity *V* vs. electrical diameter *D*; (**c**) Density plot of electrical velocity *V* vs. velocity *v*; (**d**) Density plot of pulse amplitude relative difference Δ vs. electrical diameter *D*. The fitted parabolas $D=a[1+b{\left(\Delta -c\right)}^{2}]$ are shown as red lines (average values of parameters *b* and *c*, Table 1); (**e**) Density plot of pulse amplitude relative difference Δ vs. electrical velocity *V*; (**f**) Density plot of electrical *Y* vs. *y*-coordinate; (**g**) Histogram of the corrected electrical diameter D−corr . Fitting a Gaussian allows the coefficients of variation (CVs) to be calculated as follows: 3.3%, 1.6%, and 1.4%, for the 5, 6, and 7 μ m diameter beads, respectively; (**h**) Density plot of electrical velocity *V* vs. corrected electrical diameter D−corr ; (**i**) Density plot of corrected electrical diameter D−corr vs. diameter *d*.

The electrical diameter *D* and the electrical velocity *V* cannot resolve the three bead populations, even using the novel wiring scheme (Figure 6a,b). However, the spread in estimated particle diameter *D* is reduced with respect to the conventional wiring scheme (cf. Figure 5a and Figure 6a). Moreover, the electrical velocity *V* turns out to be an excellent estimate of particle velocity *v* (Figure 6c, correlation coefficient 0.99). In fact, the peak-to-peak distance *L* of signal traces hardly depends on particle trajectory (cf. Figure 2d), and closely match the electrode pitch.

The density plots of electrical velocity *V* versus electrical diameter *D* reported in Figure 5b and Figure 6b exhibit peculiar shapes (one for each bead population). They depend on the combined effects of positional dependence of electrical diameter and velocity distribution inside the channel. Moreover, when using the conventional wiring scheme, a positional dependence of electrical velocity also comes into play. In order to gain insight into this feature, an in-depth analysis of the mapping of bead trajectories onto the DV -, DY -, and YV -planes is reported in Appendix B.

The proposed wiring scheme allows one to extract an additional metric—the pulse amplitude relative difference Δ (Equation (4)). As demonstrated in Figure 6d, the three bead populations are separated in the density plot of Δ against the electrical diameter *D*. In fact, three parabolic-shaped clusters are clearly identifiable that respectively correspond to the 5, 6, and 7 μ m diameter beads (respectively from left to right). Each cluster can be fitted to a quadratic function:

(8) $$D=a[1+b{\left(\Delta -c\right)}^{2}],$$

where *a* is particle nominal diameter, and the constants *b* and *c* account for the variation in signal with particle height as determined from the metric Δ . The fitting values of those parameters are listed in Table 1. The constant *c* should vanish due to channel symmetry top to bottom, and its fitting values are in fact negligibly small. The constants *b* and *c* should be independent of particle sizes, which is clear from Table 1, where the differences are minor. The mean value for constants *b* and *c* was then used to calculate the parabolas shown in Figure 6d for the three different particle sizes, showing an excellent fit with the data.

Equation (8) was used to correct the raw data as follows:

(9) $$D-\mathrm{corr}=\frac{D}{1+b{\left(\Delta -c\right)}^{2}},$$

where *b* and *c* are the mean values of the constants in Table 1. The corrected data is plotted in Figure 6g, showing an almost perfect Gaussian distribution. Fitting a Gaussian allows the CVs to be calculated as follows: 3.3%, 1.6%, and 1.4%, for the 5, 6, and 7 μ m diameter beads, respectively. This can be compared with the actual values of 2.5%, 1%, and 1%.

Comparing Figure 6b with Figure 6h, it appears that the simple compensation procedure in Equation (9) enabled by the proposed wiring scheme eliminates the height-dependent variation in electrical diameter *D*; i.e. all particles of a given size range have the same electrical diameter D−corr , irrespective of trajectory through the channel. The density plot of D−corr against particle diameter *d* is reported in Figure 6i. The correlation coefficient turns out to be 0.98.

A further asset of the proposed wiring scheme is its ability to supply an electrical estimate *Y* of particle trajectory *y*-coordinate by proper scaling of the metric Δ (Equation (5), H=28 μ m, α=0.75 ). This is suggested by the density plot of Δ against the electrical velocity *V* (Figure 6e), showing parabolic shapes typical of a laminar flow. The density plot of *Y* against the trajectory *y*-coordinate is reported in Figure 6f. The correlation coefficient turns out to be 0.98.

Analogous results are obtained using the coplanar electrode chip. They are shown in Figure S2, Figure S3, and Table S1 of Supplementary Materials.

## 5. Conclusions

The idea investigated via simulation in this work is as simple as it is effective: by a straightforward change in the electrical connections (the swapping of two wires), the standard microfluidic impedance chip achieves excellent performance without any particle focusing mechanisms. In particular, a pretty good estimate of particle velocity and a novel metric encoding particle trajectory are obtained from the signal traces. The new metric is exploited to obtain an accurate estimate of particle diameter, overcoming the positional dependence issue. In case of non-spherical particles (e.g., erythrocytes, budding yeasts, some blurring in estimated particle properties would remain, because particle orientation also comes into play. With respect to multi-electrode approaches [16,17], the use of a standard impedance chip equipped with just two electrode pairs reduces the volume of the sensing region, and therefore reduces the coincidence issue, enabling higher throughput. The use of a differential measurement—as in the conventional wiring scheme—attenuates the electrode polarization disturbance. Both facing and coplanar electrode designs are effective, and they could also be implemented on the same chip in order to achieve 3D particle localization. As a general design consideration, smaller channel geometries yield higher signal-to-noise ratio, but they are more prone to clogging issues. The best compromise must be identified according to the application at hand.

## Figures and Tables

**Table 1:** Facing electrode chip. Parameters of quadratic model equation $D=a[1+b{\left(\Delta -c\right)}^{2}]$ fitted to data plotted in Figure 6d.

*d* ( μ m)	*a* ( μ m)	*b*	*c*
5.0	5.01	0.90	−0.001
6.0	6.00	0.90	−0.001
7.0	7.00	0.85	−0.001
Mean	–	0.89	−0.001

**Figure A1:**
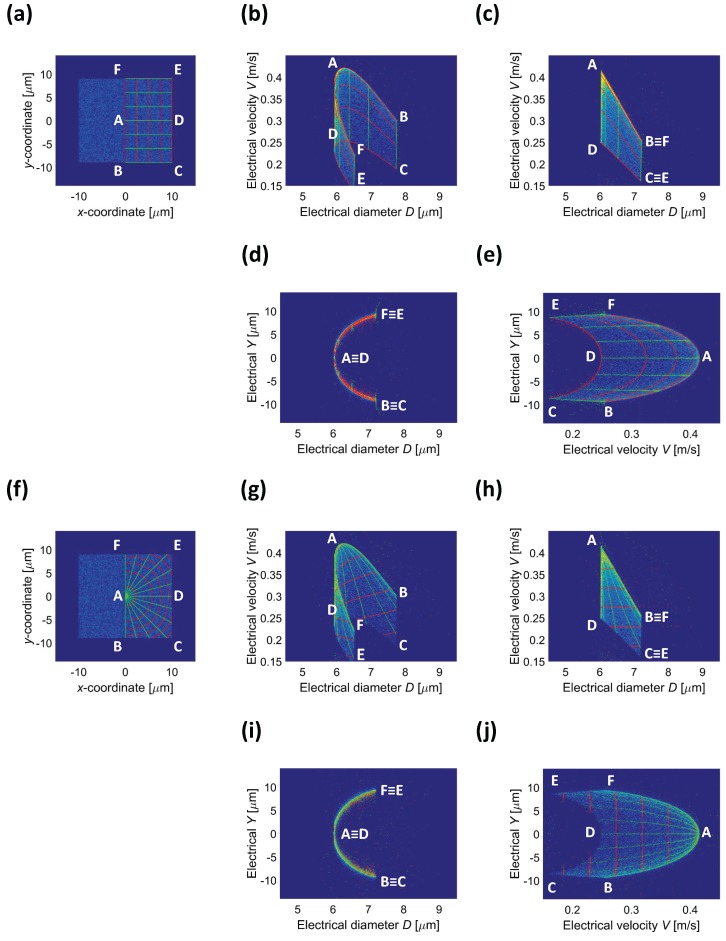
Facing electrode chip. (**a**,**f**) Density plot of *x*- and *y*-coordinates of event centers (uniformly distributed in the channel cross-section, allowing a 2 μ m gap of the 6 μ m diameter beads from the microchannel walls). Additional events along (**a**) iso-*x* and iso-*y* lines, or (**f**) iso-*v* and iso- θ lines, are marked in red and green, respectively. Six significant positions are labeled with letters from A to F. (**b**,**g**) Density plots of electrical velocity *V* vs. electrical diameter *D* relevant to particle distributions in (**a**,**d**), obtained using the conventional wiring scheme (data stream $F_{6}^{\mathrm{conv}}$ ). (**c**,**h**) Density plots of electrical velocity *V* vs. electrical diameter *D* obtained using the new wiring scheme (data stream $F_{6}^{\mathrm{new}}$ ). The latter also yields (**d**,**i**) density plots of electrical height *Y* vs. electrical diameter *D*, and (**e**,**j**) density plots of electrical height *Y* vs. electrical velocity *V*.

## Supplementary Materials

Supplementary material associated with this article (Figures S1–S4 and Table S1) can be found online at www.mdpi.com/2072-666X/8/9/283/s1, Figure S1: Fitting accuracy of the asymmetric bipolar Gaussian template (Equation (1)) to the simulated traces (suspensions of 5, 6 and 7 μ m diameter beads measured separately using the proposed wiring scheme), relevant to (a) facing electrode chip and (b) coplanar electrode chip. The histogram of the root mean squared error of the fit (RMSE), normalized by the mean value of the pulse amplitude control, $(a_{1}+a_{2})/2$ , is plotted, showing good fitting accuracy of the template, Figure S2: Coplanar electrode chip operated using the conventional wiring scheme. Virtual experiment relevant to a mixture of insulating beads with 5, 6 and 7 μ m diameter (data stream $C_{\mathrm{mix}}^{\mathrm{conv}}$ ). (a) Histogram of the electrical diameter *D*. (b) Density plot of electrical velocity *V* vs. electrical diameter *D*. (c) Density plot of electrical velocity *V* vs. velocity *v* (correlation coefficient 0.66), Figure S3: Coplanar electrode chip operated using the new wiring scheme. Virtual experiment relevant to a mixture of insulating beads with 5, 6 and 7 μ m diameter (data stream $C_{\mathrm{mix}}^{\mathrm{new}}$ ). (a) Histogram of the electrical diameter *D*. (b) Density plot of electrical velocity *V* vs. electrical diameter *D*. (c) Density plot of electrical velocity *V* vs. velocity *v* (correlation coefficient 0.96). (d) Density plot of pulse amplitude relative difference Δ vs. electrical diameter *D*. The fitted parabolas $D=a[1+b{\left(\Delta -c\right)}^{2}]$ are shown as red lines (average values of parameters *b* and *c*, Table S1). (e) Density plot of pulse amplitude relative difference Δ vs. electrical velocity *V*. (f) Density plot of electrical *X* (Equation (7), with W=40 μ m, β=0.5 ) vs. *x*-coordinate (correlation coefficient 0.97). (g) Histogram of the corrected electrical diameter D−corr . Fitting a Gaussian allows the coefficients of variation (CVs) to be calculated as follows: 4.3%, 2.4%, and 1.8%, for the 5, 6, and 7 μ m diameter beads respectively. (h) Density plot of electrical velocity *V* vs. corrected electrical diameter D−corr . (i) Density plot of corrected electrical diameter D−corr vs. diameter *d* (correlation coefficient 0.96), Figure S4: Coplanar electrode chip. (a,f) Density plot of *x*- and *y*-coordinates of event centers (uniformly distributed in the channel cross-section, allowing a 2 μ m gap of the 6 μ m diameter beads from the microchannel walls). Additional events along (a) iso-*x* and iso-*y* lines, or (f) iso-*v* and iso- θ lines, are marked in red and green, respectively. Six significant positions are labeled with letters from A to F. (b,g) Density plots of electrical velocity *V* vs. electrical diameter *D* relevant to particle distributions in (a,d), obtained using the conventional wiring scheme (data stream $C_{6}^{\mathrm{conv}}$ in Table A1). (c,h) Density plots of electrical velocity *V* vs. electrical diameter *D* obtained using the new wiring scheme (data stream $C_{6}^{\mathrm{new}}$ in Table A1). The latter also yields (d,i) density plots of electrical position *X* vs. electrical diameter *D*, and (e,j) density plots of electrical position *X* vs. electrical velocity *V*, Table S1: Coplanar electrode chip. Parameters of quadratic model equation $D=a[1+b{\left(\Delta -c\right)}^{2}]$ used to fit data plotted in Figure S3(d).

## Appendix A. Synthetic Data Streams Generation in a Virtual Laboratory

### Appendix A.1. Data Streams

The generic data stream is relevant to a mixture of $N_{p}$ populations of dielectric spherical beads suspended in a conductive buffer at respective concentrations $\rho_{1},\ldots ,\rho_{N_{p}}$ , pumped through the device at a flow rate ϕ . Population nominal diameters are $d_{1},\ldots ,d_{N_{p}}$ , with coefficients of variation ${\mathrm{CV}}_{1},\ldots ,{\mathrm{CV}}_{N_{p}}$ , respectively.

A number $N_{e}$ of events (i.e., passage of a particle in the sensing region) is generated. The typical event *e* is characterized by the following quantities:

- $p_{e}\in \left\{1,\ldots ,N_{p}\right\}$ : population index, denoting the population the event belongs to, drawn from the finite sample space $\left\{1,\ldots ,N_{p}\right\}$ with the probabilities $\rho_{1}/\rho ,\ldots ,\rho_{N_{p}}/\rho$ , where $\rho =\sum_{p=1}^{N_{p}}\rho_{p}$ is the total particle concentration;
- $d_{e}$ : particle diameter, drawn from the Gaussian distribution with mean $d_{p_{e}}$ and standard deviation $\sigma_{p_{e}}=\mathrm{CV}{\;}_{p_{e}}\;d_{p_{e}}$ ;
- $\left(x_{e},y_{e}\right)$ : (x,y) -coordinates of the particle trajectory in the channel cross-section, drawn from a uniform distribution in the available cross-section region (a 2 μ m gap between particle boundary and channel walls has been assumed);
- $v_{e}$ : particle velocity, determined as a function of $\left(x_{e},y_{e}\right)$ assuming laminar flow [26] (in fact, Reynolds number is typically in the order of units);
- $t_{e}$ : particle entrance time (i.e., time instant the particle center passes through the entrance cross-section). Occurrence of particles was assumed to be a Poisson process [18]. Accordingly, particle inter-arrival times $\Delta t_{e}$ were drawn from an exponential distribution with rate parameter λ=ϕρ .

Assuming that particles do not interact with each other, the signal trace S(t) —measuring the differential current $I_{\mathrm{Diff}}$ —can be obtained by adding the contributions of the events with entrance time $t_{e}\leq t$ :

(A1)
$$S\left(t\right)=\sum_{\left\{e\;:\;t\geq t_{e}\right\}}S_{p_{e}}\left(x_{e},y_{e},z_{e}\left(t\right)\right)\;{\left(\frac{d_{e}}{d_{p_{e}}}\right)}^{3}.$$

Because particles essentially experience uniform linear motion in the microchannel (at least over distances of the order of the sensing region length), the law of motion $z_{e}\left(t\right)=v_{e}\left(t-t_{e}\right)$ can be assumed. In case of the facing electrode (respectively, coplanar electrode) chip, the function $S_{p_{e}}\left(x_{e},y_{e},z_{e}\left(t\right)\right)$ is independent of $x_{e}$ (respectively, $y_{e}$ ), because the electric field is homogeneous along the *x*-axis (respectively, *y*-axis). Its value at $\left(y_{e},z_{e}\left(t\right)\right)$ (respectively, $\left(x_{e},z_{e}\left(t\right)\right)$ ) is obtained by means of 2D interpolation of a repository of pre-computed finite element simulation values over a regular grid of (y,z) (respectively, (x,z) ) locations, for each nominal population diameter (e.g., Figure 2b,d). Finally, the factor ${\left(d_{e}/d_{p_{e}}\right)}^{3}$ in Equation (A1) accounts for the actual particle diameter, which is normally distributed around the nominal population diameter. White noise with standard deviation $\sigma_{N}$ was added to the data stream. A filter consisting of *n* first-order filtering steps was implemented, with resulting filter bandwidth BW . A sampling frequency $f_{s}$ was assumed.

The parameter values used in the generation of the synthetic data streams considered in this work are reported in Table A1. The one relevant to the mixture of beads is typical of experimental settings (e.g., [16,17]). Ideal noise-free data streams are also generated in order to investigate the mapping of bead trajectories onto the DV -, DY -, and YV -planes (Appendix B).

**Table A1:** Parameter values used in the generation of the synthetic data streams (for all data streams: ϕ=10 μ L/min, n=4 , BW=20 kHz, $f_{s}=115$ ksps).

	Layout	Wiring	$N_{p}$	$d_{1-3}$ [ μ m]	${\mathrm{CV}}_{1-3}\;\left[\%\right]$	$\rho_{1-3}\;$ [#/ μ L]	$\sigma_{N}$ [nA]
$F_{\mathrm{mix}}^{\mathrm{conv}}$	facing	conventional	3	5, 6, 7	2.5, 1, 1	$10^{3}/3$	8.4
$F_{\mathrm{mix}}^{\mathrm{new}}$	facing	novel	3	5, 6, 7	2.5, 1, 1	$10^{3}/3$	8.4
$C_{\mathrm{mix}}^{\mathrm{conv}}$	coplanar	conventional	3	5, 6, 7	2.5, 1, 1	$10^{3}/3$	7.0
$C_{\mathrm{mix}}^{\mathrm{new}}$	coplanar	novel	3	5, 6, 7	2.5, 1, 1	$10^{3}/3$	7.0
$F_{6}^{\mathrm{conv}}$	facing	conventional	1	6	0	$10^{3}$	0
$F_{6}^{\mathrm{new}}$	facing	novel	1	6	0	$10^{3}$	0
$C_{6}^{\mathrm{conv}}$	coplanar	conventional	1	6	0	$10^{3}$	0
$C_{6}^{\mathrm{new}}$	coplanar	novel	1	6	0	$10^{3}$	0

### Appendix A.2. Finite Element Model Equations

Model equations have been described elsewhere (e.g., [27,28]), and are summarized here for the sake of completeness. The device is modeled as the union of two homogeneous conducting regions $\Omega_{p}$ and $\Omega_{b}$ , representing the particle and the fluid buffer, respectively. Their complex conductivities $\sigma_{p}^{*}$ and $\sigma_{b}^{*}$ are given by $\sigma_{k}^{*}=\sigma_{k}+i\omega \varepsilon_{k}\varepsilon_{v}$ , k∈{p,b} , where $\varepsilon_{v}$ is the permittivity of free space, and $\sigma_{k}$ and $\varepsilon_{k}$ are the conductivity and relative permittivity of the media, respectively; ω denotes the circular frequency, and i is the imaginary unit. Continuity of electric potential and of normal current flux density is enforced at the particle surface Γ . The boundary of the domain is divided into an insulating part ( $\partial \Omega_{ne}$ ), and a part covered by electrodes ( $\partial \Omega_{e}$ ).

In the Fourier domain, the electrical problem is stated as follows:

(A2)−div(σ*∇Ψ)=0,inΩp∪Ωb;(A3)[[σ*∇Ψ·n]]=0,onΓ;(A4)[[Ψ]]=0,onΓ,

where Ψ is the electric potential phasor, $\sigma^{*}=\sigma_{k}^{*}$ in $\Omega_{k}$ , k∈{p,b} , div and *∇* respectively denote the divergence and gradient operators, [[·]] is the jump of the enclosed quantity across Γ , and n denotes the outer unit normal vector. An insulating boundary condition is applied on the boundaries not covered by electrodes:

(A5)
$$\sigma_{b}^{*}\nabla \Psi \cdot n=0\;,\;\mathrm{on}\partial \Omega_{ne}\;.$$

On the *i*-th electrode ( $\partial \Omega_{e_{i}}$ ), the following electrode equation holds:

(A6)
$$Y_{e}\left(\Psi_{i}-\Psi \right)=\sigma_{b}^{*}\nabla \Psi \cdot n\;,\;\mathrm{on}\partial \Omega_{e_{i}}\;,$$

where $Y_{e}=G_{e}+i\omega C_{e}$ is the double-layer admittance per unit area, expressed in terms of conductance $G_{e}$ and capacitance $C_{e}$ per unit area, and $\Psi_{i}$ is the electrode potential. The inward current flowing through electrode *i* is given by:

(A7)
$$I_{i}=\int_{\partial \Omega_{e_{i}}}\sigma_{b}^{*}\nabla \Psi \cdot n\;dA,\;\mathrm{on}\partial \Omega_{e_{i}}\;.$$

Parameter values used in the simulations are relevant to the experimental setup described in the companion experimental paper [19], and are reported in Table A2.

**Table A2:** Parameter values used in the finite element simulations.

ω (rad/s)	$C_{e}$ (mF/m ${}^{2}$ )	$\sigma_{b}$ (S/m)	$\varepsilon_{b}/\varepsilon_{v}$	$\sigma_{p}$ (S/m)	$\varepsilon_{p}/\varepsilon_{v}$
2 $\pi \times 10^{6}$	33	1.1	80	$6.6\times 10^{-4}$	2.5

Quadratic Lagrangian tetrahedral elements were used to interpolate the electric potential Ψ . The typical mesh involved about 100,000 tetrahedral elements and 150,000 degrees of freedom. The computational time required for the computation of the differential current $I_{\mathrm{Diff}}$ for one *z*-position was about 30 s on a workstation equipped with Intel(R) Xeon(R) CPU processor E5-2660 v3 @ 2.60 GHz (Intel, Santa Clara, CA, USA) and 128 GB RAM (Corsair Components, Fremont, CA, USA).

## Appendix B. Mapping of Bead Trajectories onto the *DV*-, *DY*-, and *YV*-Planes

In order to gain insight into the relationship between bead trajectory position in the channel cross-section and corresponding electrical estimates (*D*, *V*, and *Y*), noise-free data streams relevant to a single population of 6 μ m beads with identical diameter (i.e., vanishing CV) were generated ( $F_{6}^{\mathrm{conv}}$ , $F_{6}^{\mathrm{new}}$ in Table A1). They were used to construct the 2D mapping which associates a point (D,V) to every bead trajectory, identified by the (x,y) -coordinates of bead center in the channel cross-section (Figure A1). The image of this mapping is a region R in the (D,V) -plane (Figure A1b,c,g,h).

Besides events produced by beads uniformly distributed in the channel cross-section (Appendix A), auxiliary events were generated, produced by beads whose centers have (x,y) -coordinates distributed along two grids: (i) a Cartesian grid with iso-*x* and iso-*y* lines (Figure A1a); and (ii) a grid comprising iso-*v* and iso- θ lines (Figure A1f), where θ denotes the polar angle. The iso-*v* lines have an approximately elliptical shape, according to the velocity distribution in steady state, hydrodynamically fully developed, laminar flow for Newtonian fluids in rectangular channels [26]. Only one half of the channel in Figure A1a,f is covered by grids, due to symmetry with respect to the *y* axis. Six significant positions are labeled with letters from A to F.

Using the proposed wiring scheme, the new metric Δ is available, yielding the electric estimate *Y* of particle trajectory height *y*. Hence, a mapping from the (x,y) plane onto a surface in the (D,V,Y) space is in fact obtained. Besides the classical projection of that map onto the (D,V) plane introduced above, projections onto the (D,Y) and (V,Y) planes are also available.

The analysis of Figure A1 reveals that:

- iso-*y* lines (Figure A1a) are mapped onto iso-*D* lines (Figure A1b,c). In fact, the electric field is homogeneous along the *x*-axis, such that different *x* values yield the same value of *D*; on the other hand, the quotient map y→D just defines the positional dependence issue addressed in this paper (the higher the distance from the channel axis, the higher *D*). A top–bottom asymmetry is observed when using the conventional wiring scheme, whereas symmetry top-to-bottom is obtained using the novel wiring scheme (e.g., top and bottom iso-*y* lines BC and FE overlap and are mapped on the right straight line of the boundary of R (Figure A1c). Moreover, iso-*y* lines are mapped onto iso-*Y* lines (Figure A1d,e), confirming that *Y* is a suitable estimate of *y*;
- iso-*v* lines (Figure A1f) are mapped onto iso-*V* lines using the novel wiring scheme (cf. Figure A1h,j), confirming that the novel wiring scheme relieves the positional dependence issue of the electrical velocity *V* exhibited by the conventional wiring scheme (Figure A1g);
- iso-*x* lines (Figure A1a) are mapped onto curved contours (Figure A1b) or straight lines (Figure A1c). The closer to the center the iso-*x* line, the higher *V*, the higher is the contour; peak velocity on BAF (respectively, CDE) corresponds to central trajectory A (respectively, D) yielding the smallest electric diameter *D*. Moreover, iso-*x* lines are mapped onto parabolic profiles in the (Y,V) plane (Figure A1e), typical of laminar flow;
- the top contour of R (Figure A1g,h) is the image of the θ=±π/2 isolines (Figure A1f); the branches AB and AF overlap using the proposed wiring scheme (Figure A1h) due to symmetry top to bottom. The left contour of R is the image of the θ=0 isoline AD, because particles equidistant from top and bottom electrodes have the smallest electrical diameter *D*.

The projection onto the (D,Y) plane (Figure A1d,i) approximately reduces to a line (cf. also Figure 6d). This is the reason why the simple compensation procedure in Equation (9) is effective.

Analogous results shown in Figure S4 of Supplementary Materials are obtained with the coplanar electrode chip, the role played by the *x*- and *y*-coordinate being swapped with respect to the facing electrode chip.

The insight gained by this analysis could be very helpful in interpreting experimental results involving, e.g., passive or active particle focusing mechanisms.

## References

[1] Sun T., Morgan H. (2010). Single-cell microfluidic impedance cytometry: A review. Microfluid. Nanofluid..

[2] Petchakup C., Li K.H.H., Hou H.W. (2017). Advances in Single Cell Impedance Cytometry for Biomedical Applications. Micromachines.

[3] McGrath J.S., Honrado C., Spencer D., Horton B., Bridle H.L., Morgan H. (2017). Analysis of Parasitic Protozoa at the Single-cell Level using Microfluidic Impedance Cytometry. Sci. Rep..

[4] Xie X., Cheng Z., Xu Y., Liu R., Li Q., Cheng J. (2017). A sheath-less electric impedance micro-flow cytometry device for rapid label-free cell classification and viability testing. Anal. Methods.

[5] Mansor M., Takeuchi M., Nakajima M., Hasegawa Y., Ahmad M.R. (2017). A Novel Integrated Dual Microneedle-Microfluidic Impedance Flow Cytometry for Cells Detection in Suspensions. Int. J. Electr. Comput. Eng..

[6] Esfandyarpour R., DiDonato M.J., Yang Y., Durmus N.G., Harris J.S., Davis R.W. (2017). Multifunctional, inexpensive, and reusable nanoparticle-printed biochip for cell manipulation and diagnosis. Proc. Natl. Acad. Sci. USA.

[7] Gawad S., Cheung K., Seger U., Bertsch A., Renaud P. (2004). Dielectric spectroscopy in a micromachined flow cytometer: Theoretical and practical considerations. Lab Chip.

[8] Cheung K.C., Di Berardino M., Schade-Kampmann G., Hebeisen M., Pierzchalski A., Bocsi J., Mittag A., Tárnok A. (2010). Microfluidic impedance-based flow cytometry. Cytom. Part A.

[9] Sun T., Green N.G., Gawad S., Morgan H. (2007). Analytical electric field and sensitivity analysis for two microfluidic impedance cytometer designs. IET Nanobiotechnol..

[10] Spencer D., Morgan H. (2011). Positional dependence of particles in microfludic impedance cytometry. Lab Chip.

[11] Errico V., De Ninno A., Bertani F.R., Businaro L., Bisegna P., Caselli F. (2017). Mitigating positional dependence in coplanar electrode Coulter-type microfluidic devices. Sens. Actuators B Chem..

[12] Clausen C.H., Skands G.E., Bertelsen C.V., Svendsen W.E. (2015). Coplanar Electrode Layout Optimized for Increased Sensitivity for Electrical Impedance Spectroscopy. Micromachines.

[13] Rollo E., Tenaglia E., Genolet R., Bianchi E., Harari A., Coukos G., Guiducci C. (2017). Label-free identification of activated T-lymphocytes through tridimensional microsensors on chip. Biosens. Bioelectron..

[14] Shaker M., Colella L., Caselli F., Bisegna P., Renaud P. (2014). An impedance-based flow micro-cytometer for single cell morphology discrimination. Lab Chip.

[15] Grenvall C., Antfolk C., Bisgaard C., Laurell T. (2014). Two-dimensional acoustic particle focusing enables sheathless chip Coulter counter with planar electrode configuration. Lab Chip.

[16] Spencer D., Caselli F., Bisegna P., Morgan H. (2016). High accuracy particle analysis using sheathless microfluidic impedance cytometry. Lab Chip.

[17] De Ninno A., Errico V., Bertani F.R., Businaro L., Bisegna P., Caselli F. (2017). Coplanar electrode microfluidic chip enabling accurate sheathless impedance cytometry. Lab Chip.

[18] Hassan U., Bashir R. (2014). Coincidence detection of heterogeneous cell populations from whole blood with coplanar electrodes in a microfluidic impedance cytometer. Lab Chip.

[19] Caselli F., De Ninno A., Reale R., Businaro L., Bisegna P. (2017). A novel wiring scheme for standard chips enabling high-accuracy impedance cytometry.

[20] Sun T., van Berkel C., Green N.G., Morgan H. (2009). Digital signal processing methods for impedance microfluidic cytometry. Microfluid. Nanofluid..

[21] Caselli F., Bisegna P. (2016). A simple and robust event-detection algorithm for single-cell impedance cytometry. IEEE Trans. Biomed. Eng..

[22] Demierre N., Braschler T., Linderholm P., Seger U., van Lintel H., Renaud P. (2007). Characterization and optimization of liquid electrodes for lateral dielectrophoresis. Lab Chip.

[23] Mernier G., Duqi E., Renaud P. (2012). Characterization of a novel impedance cytometer design and its integration with lateral focusing by dielectrophoresis. Lab Chip.

[24] Gawad S., Schild L., Renaud P. (2001). Micromachined impedance spectroscopy flow cytometer for cell analysis and particle sizing. Lab Chip.

[25] Caselli F., Bisegna P. (2017). Simulation and performance analysis of a novel high-accuracy sheathless microfluidic impedance cytometer with coplanar electrode layout. Med. Eng. Phys..

[26] Spiga M., Morini G.L. (1994). A symmetric solution for velocity profile in laminar flow through rectangular ducts. Int. Commun. Heat. Mass.

[27] Caselli F., Bisegna P., Maceri F. (2010). EIT-Inspired Microfluidic Cytometer for Single-Cell Dielectric Spectroscopy. J. Microelectromech. Syst..

[28] Caselli F., Shaker M., Colella L., Renaud P., Bisegna P. (2014). Modeling, Simulation and Performance Evaluation of a Novel Microfluidic Impedance Cytometer for Morphology-Based Cell Discrimination. J. Microelectromech. Syst..

