# Supplementary material for: Numerical Investigation of a Novel Wiring Scheme Enabling Simple and Accurate Impedance Cytometry
Source: Micromachines (Basel). 2017 Sep 18;8(9):283. doi: 10.3390/mi8090283 (PMC6190262; doi:10.3390/mi8090283)
Supplement: Supplementary file 1 [file micromachines-08-00283-s001.pdf]

Supplementary Materials: Numerical Investigation of a Novel Wiring Scheme Enabling Simple and Accurate Impedance Cytometry

Federica Caselli *, Riccardo Reale, Nicola Antonio Nodargi and Paolo Bisegna *

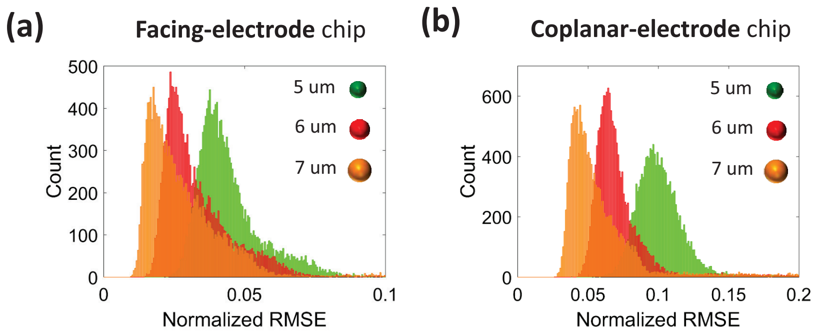

Figure S1. Fitting accuracy of the asymmetric bipolar Gaussian template (Equation (1) of the main text) to the simulated traces (suspensions of 5, 6 and 7 μm diameter beads measured separately using the proposed wiring scheme), relevant to (**a**) facing electrode chip and (**b**) coplanar electrode chip. The histogram of the root mean squared error of the fit (RMSE), normalized by the mean value of the pulse amplitude control, $(a_1 + a_2)/2$, is plotted, showing good fitting accuracy of the template.

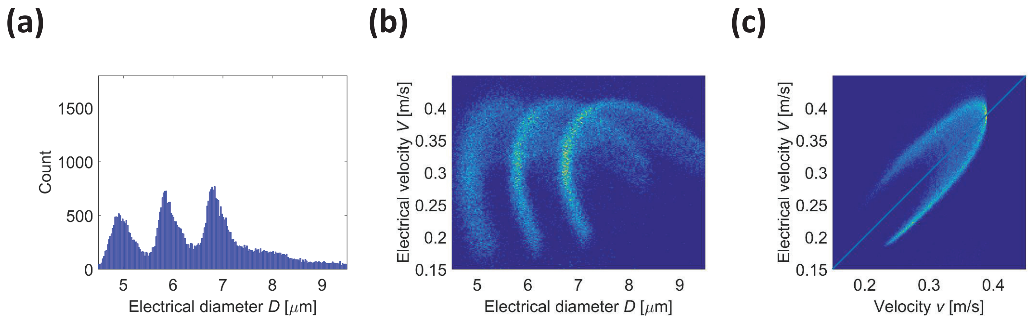

Figure S2. Coplanar electrode chip operated using the conventional wiring scheme. Virtual experiment relevant to a mixture of insulating beads with 5, 6 and 7 μm diameter (data stream $\mathcal{C}_{\text{mix}}^{\text{conv}}$). (**a**) Histogram of the electrical diameter D; (**b**) Density plot of electrical velocity V vs electrical diameter D; (**c**) Density plot of electrical velocity V vs velocity v (correlation coefficient 0.66).

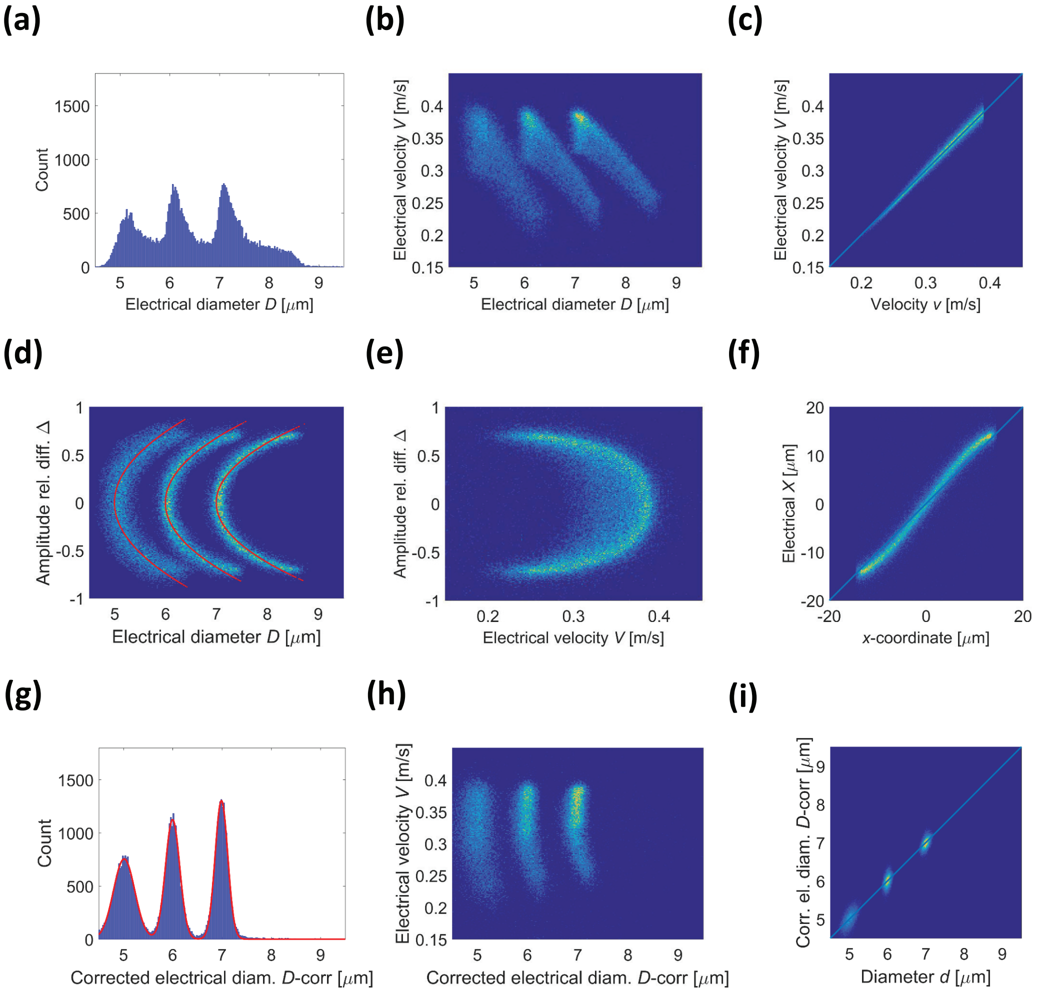

Figure S3. Coplanar electrode chip operated using the new wiring scheme. Virtual experiment relevant to a mixture of insulating beads with 5, 6 and 7 μm diameter (data stream $\mathcal{C}_{mix}^{new}$). (**a**) Histogram of the electrical diameter D; (**b**) Density plot of electrical velocity V vs electrical diameter D; (**c**) Density plot of electrical velocity V vs velocity v (correlation coefficient 0.96); (**d**) Density plot of pulse amplitude relative difference Δ vs electrical diameter D. The fitted parabolas $D = a[1 + b(\Delta - c)^2]$ are shown as red lines (average values of parameters b and c, Table S1); (**e**) Density plot of pulse amplitude relative difference Δ vs electrical velocity V; (**f**) Density plot of electrical X (Equation (7) of the main text, with $W = 40$ μm, $\beta = 0.5$) vs x-coordinate (correlation coefficient 0.97); (**g**) Histogram of the corrected electrical diameter D-corr. Fitting a Gaussian allows the coefficients of variation (CVs) to be calculated as follows: 4.3%, 2.4%, and 1.8%, for the 5, 6, and 7 μm diameter beads respectively; (**h**) Density plot of electrical velocity V vs corrected electrical diameter D-corr; (**i**) Density plot of corrected electrical diameter D-corr vs diameter d (correlation coefficient 0.96).

Table S1. Coplanar electrode chip. Parameters of quadratic model equation $D = a[1 + b(\Delta - c)^2]$ used to fit data plotted in Figure S3(d).

d (μm)	a (μm)	b	c
5.0	5.02	0.35	0.002
6.0	5.99	0.38	−0.003
7.0	6.98	0.38	−0.001
Mean	–	0.37	−0.001

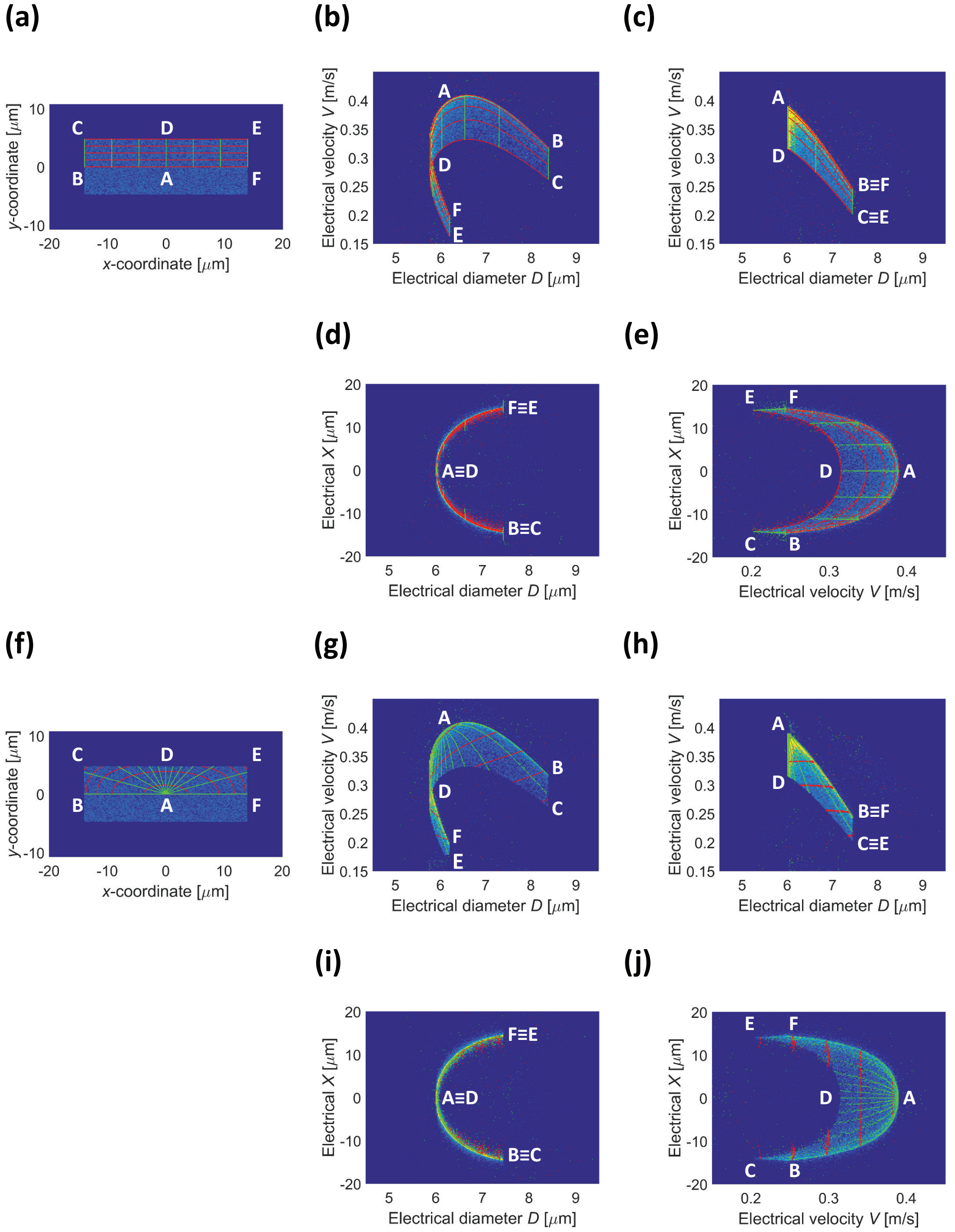

Figure S4. Coplanar electrode chip. (**a**,**f**) Density plot of x- and y-coordinates of event centers (uniformly distributed in the channel cross-section, allowing a 2 μm gap of the 6 μm diameter beads from the microchannel walls). Additional events along (**a**) iso-x and iso-y lines, or (**f**) iso-v and iso-θ lines, are marked in red and green, respectively. Six significant positions are labeled with letters from A to F. (**b**,**g**) Density plots of electrical velocity V vs electrical diameter D relevant to particle distributions in (**a**,**d**), obtained using the conventional wiring scheme (data stream $\mathcal{C}_6^{\text{conv}}$ in Table A1 of the main text). (**c**,**h**) Density plots of electrical velocity V vs electrical diameter D obtained using the new wiring scheme (data stream $\mathcal{C}_6^{\text{new}}$ in Table A1 of the main text). The latter also yields (**d**,**i**) density plots of electrical position X vs electrical diameter D, and (**e**,**j**) density plots of electrical position X vs electrical velocity V.
